# Evaluation of the Clinical Significance of Assessing Previous Gambling Problems Before Initiating Antipsychotic Treatment: An Audit

**DOI:** 10.1192/bjo.2024.567

**Published:** 2024-08-01

**Authors:** Evelyn Farthing

**Affiliations:** GMMH, Manchester, United Kingdom

## Abstract

**Aims:**

This audit aims to address the critical link between antipsychotics and impulsive behaviors, particularly pathological gambling, by emphasizing the importance of assessing patients' gambling history before initiating antipsychotic treatment. The focus is on patients under the care of the Bolton Early Intervention in Psychosis (EIT) service, with the aim of meeting the standard set by NICE guidelines, ensuring that 100% of patients started on antipsychotics are asked about their previous gambling history.

**Methods:**

Data was collected from prescription and shared care protocol lists for patients prescribed antipsychotics in the last six months. The PARIS progress notes and clinical correspondence were then searched to determine if patients had been asked about gambling.

**Results:**

The audit revealed a significant gap in the practice, with minimal adherence to NICE guidelines regarding assessing gambling history before prescribing antipsychotics. Out of 35 patients, only one was asked about gambling history.

**Conclusion:**

The recommendations for improvement include incorporating a gambling prompt into the medical review proforma, educating the team about the importance of this assessment, and adding the Problem Gambling Severity Index to the initial review by EIT.

